# Women’s preferences for HPV self-sampling in cervical cancer screening: a discrete choice experiment

**DOI:** 10.3389/fpubh.2026.1779443

**Published:** 2026-04-13

**Authors:** Ni Ding, Yunlong Chen, Xueyuan Zhou, Gang Wang, Jiaying Li, Xiaohua Wang

**Affiliations:** 1 Graduate School, Inner Mongolia Medical University, Hohhot, China; 2 Department of Thoracic Surgery, National Cancer Center/National Clinical Research Center for Cancer/Cancer Hospital, Chinese Academy of Medical Sciences and Peking Union Medical College, Beijing, China; 3 The Second Hospital of Shandong University, Shandong University, Jinan, China; 4 Department of Genetics, Inner Mongolia Maternity and Child Health Care Hospital, Hohhot, China; 5 The Nethersole School of Nursing, Faculty of Medicine, The Chinese University of Hong Kong, Hong Kong, Hong Kong SAR, China; 6 Inner Mongolia Medical University, Hohhot, China

**Keywords:** cervical cancer screening, discrete choice experiment, HPV self-sampling, preferences, public health

## Abstract

**Background:**

HPV self-sampling can increase cervical cancer screening coverage. To effectively implement this innovative screening method, it is crucial to gain an in-depth understanding of women’s acceptance and preferences regarding each stage of the HPV self-sampling intervention process. This study aims to identify the key attributes of HPV self-sampling that drive acceptance among under-screened populations, thereby informing the development of equitable screening strategies that reduce barriers and expand access to cervical cancer prevention.

**Methods:**

In this cross-sectional study, we conducted a Discrete Choice Experiment (DCE) between 1 September 2024 and 31 March 2025, assessing preferences for five attributes of HPV self-sampling: accuracy, procedural difficulty, comfort, sampling time, and price. Analyses employed a mixed logit model, adhering to the Discrete Choice Experiment Reporting Checklist (DIRECT) reporting guideline. We estimated relative importance (RI) for each attribute, derived willingness-to-pay (WTP), and examined preference heterogeneity using interaction terms.

**Results:**

Of 200 valid responses, accuracy carried the greatest weight (RI 42.55%), followed by price (20.67%), sampling time (13.66%), procedural difficulty (11.68%), and comfort (11.45%). Low accuracy markedly reduced acceptance (*β* = −6.640, *p* < 0.001), as did higher price (*β* = −0.011, *p* < 0.001) and longer sampling time (>5 min; *β* = −1.703, *p* < 0.001). Participants showed a positive preference for more difficult procedures (*β* = 1.550, *p* < 0.01), and moderate comfort increased acceptance (*β* = 1.192, *p* < 0.001). Preference heterogeneity was observed: older age was associated with less aversion to low accuracy (*β* = 0.190, *p* < 0.001), greater tolerance of procedural difficulty (*β* = 0.106, *p* < 0.01), and a higher preference for moderate comfort (*β* = 0.037, *p* < 0.05). Ethnic minorities were less averse to low accuracy than Han respondents (*β* = 3.085, *p* < 0.01). Higher education was associated with a lower preference for moderate comfort (*β* = −0.375, *p* < 0.05). Women already inclined towards self-sampling were more averse to low accuracy (*β* = −2.623, *p* < 0.001) and procedural difficulty (*β* = −1.095, *p* < 0.05).

**Conclusion:**

Accuracy, price, and sampling time are the dominant drivers of women’s choices for HPV self-sampling, and are simultaneously significantly moderated by individual characteristics such as age, ethnicity, education, and sampling preference. These findings underscore that effective screening programs must not only optimize core product attributes but also develop tailored strategies to address the distinct preferences of different demographic subgroups.

## 1 Introduction

Cervical cancer is the fourth most common cancer in women globally (1). Persistent infection with high-risk human papillomavirus (HPV) causes most cases (2). In China, the burden remains substantial: the age-standardized incidence increased at an average annual percentage change of 14.8% during 2000–08 and continued to increase, though more slowly, by 1.7% per year during 2008–18, coverage of screening is uneven, and HPV risk persists, particularly in settings with limited health resources (3). Cervical cancer screening is the cornerstone of secondary prevention, enabling early detection of HPV infection and precancerous lesions and thereby reducing progression to invasive disease (4, 5). The burden is disproportionately borne by rural and economically disadvantaged regions, where access to HPV vaccination and screening is constrained; more than 60% of cases occur in rural areas (6). As an economically underdeveloped region, the Inner Mongolia Autonomous Region faces challenges like limited screening accessibility and low participation rates among women, providing the primary rationale for conducting this research in this region (7).

Traditional screening involves clinician-collected samples for cytology and/or HPV testing, but participation can be limited by privacy concerns, travel and logistical challenges, and uneven distribution of trained staff (8–10). HPV self-sampling is a method where women collect their own vaginal sample—using a simple swab, brush, or lavage device—for subsequent high-risk HPV DNA testing in a laboratory. The procedure is typically designed to be straightforward, with pictorial instructions guiding the user. Samples can be collected in the privacy of one’s home and mailed to a lab or returned to a healthcare facility (11–15). This approach enables women to collect samples privately, avoiding the embarrassment, discomfort, and logistical challenges often associated with clinician-performed pelvic examinations (12, 13). By removing these barriers, self-sampling has been shown to effectively reach under-screened populations (11). A systematic review and meta-analysis confirmed that offering self-sampling nearly doubles the probability of cervical cancer screening uptake among under-screened women (14). Consequently, an increasing number of countries are incorporating self-sampling into their national screening programs, either for under-screened populations or as a primary option for all eligible women, to enhance accessibility and reduce disparities in screening coverage (15).

This method has been shown to have comparable accuracy to clinician-collected samples (16–20). Beyond accuracy, evidence also supports its favorable cost-effectiveness and high acceptance, particularly in resource-limited settings. For instance, a cost-utility analysis from Thailand found that a screening policy combining self-collected and clinician-collected samples for HPV DNA testing was the dominant strategy, providing greater health benefits at lower costs compared to clinician-collected sampling alone, with the additional benefit achievable at any increased screening rate (16). In the United States, a recent clinical trial validating a self-collection device designed for at-home use reported high positive percent agreement (95.2%) with clinician-collected samples for detecting high-risk HPV and equivalent clinical sensitivity for cervical dysplasia, with 93% of participants indicating they would prefer self-sampling if results were comparable (17). A systematic review of values and preferences across diverse settings confirmed that self-sampling is highly acceptable, with women valuing its ease of use, convenience, privacy, and comfort (18). In Zimbabwe, a discrete choice experiment (DCE) among rural women identified the comfort of the sampling device as the most significant factor influencing preferences for HPV self-sampling, underscoring the importance of user-centered device design in resource-limited contexts (19). Furthermore, a community-based pilot study in rural Ethiopia demonstrated the feasibility of home-based HPV self-sampling, achieving a screening acceptance rate of 85% among eligible women, illustrating the potential of this approach to reach hard-to-access populations in low-income countries (20).

The successful implementation and uptake of HPV self-sampling are highly dependent on women’s preferences for its specific attributes. A growing body of literature has employed DCE and acceptability studies to quantify these preferences globally. Studies from diverse settings, such as South Africa (21), Zambia (22), and the United Kingdom (23), have identified key determinants of choice, including cost, accuracy, convenience, and privacy. In addition to these quantitative DCEs, evidence from general acceptability studies across diverse income settings further confirms that self-sampling is highly acceptable, with women valuing its privacy, convenience, and comfort compared to clinician-collected samples (18, 24, 25).

Within China, research on attitudes towards HPV self-sampling is also emerging. Studies in Jiangsu, Zhejiang, and Shanghai have demonstrated high acceptability and a preference for self-sampling among many women, linking this preference to factors such as convenience and confidence in performing the procedure (26). Similarly, investigations in Yunnan province highlighted high comfort levels with and a preference for self-sampling among ethnically diverse women, underscoring its potential to reach underserved populations (27). Furthermore, a recent randomized controlled trial protocol in Hohhot, Inner Mongolia, explores innovative strategies like the “pay-it-forward” model to improve the feedback rate of self-sampling results, indicating a growing interest in implementing this method in the region (28).

Despite these valuable contributions, a critical evidence gap persists concerning Northern China. While the aforementioned studies provide valuable insights into general acceptability, there is a stark lack of quantitative evidence from rigorous preference-elicitation methods like DCEs to understand the specific trade-offs that under-screened women in Northern China, particularly in socio-economically and geographically diverse regions like Inner Mongolia, are willing to make. Existing research from other Chinese provinces may not be generalizable to this context due to unique regional disparities in healthcare access, cultural norms, and economic conditions. A precise understanding of the relative importance women assign to core attributes is therefore indispensable for designing a screening service that will achieve high uptake. The present study addresses this identified gap. We conducted a DCE in Hohhot. As a representative urban center in Northern China, Hohhot serves as a relevant setting for this investigation. While it functions as the capital of the Inner Mongolia Autonomous Region, it still reflects broader regional challenges such as geographic barriers, disparities in healthcare access, and under-screening among vulnerable groups. These characteristics make it a pertinent context for studying the potential of self-sampling to improve cervical cancer prevention.

DCEs, grounded in random utility theory, quantify preferences for multi-attribute health services and their relative importance (29–31). The core design of the experiment is to establish a set of attributes and levels to construct a virtual selection environment. In this environment, the experimenter hypothesizes that the decision maker will systematically evaluate the attributes of the options and then make a value comparison based on the principle of utility maximization. Ultimately, the experiment will generate a selection result that aligns with the probability distribution (32). Given the scarcity of empirical research on women’s preferences regarding HPV self-sampling in Inner Mongolia (33), a method capable of capturing nuanced preferences and informing context-specific policy is essential. DCE was selected for this study because it allows for the systematic elicitation of preferences among attributes such as sampling accuracy, convenience, cost, and procedural difficulty, each critical to the design of appropriate and accessible cervical screening programs.

Therefore, this study employed a DCE in Hohhot to fill this critical evidence gap. It aims to quantitatively determine the key drivers of choices regarding HPV self-sampling among under-screened women and to estimate the relative value they place on attributes such as accuracy, cost, and procedural characteristics. Beyond informing context-specific screening strategies for Inner Mongolia, the findings will contribute a methodological framework for quantifying patient preferences in underserved settings. This evidence is vital for designing equitable, acceptable, and effective cervical cancer screening programs that can truly reach populations most in need, both within China and in similar global contexts.

## 2 Materials and methods

### 2.1 Study design

DCEs are a quantitative research method designed to measure respondents’ preferences. Respondents are presented with repeated hypothetical scenarios (termed choice tasks), each characterized by the same attributes but differing levels, and typically containing two to three options (34). Through their selection of the preferred option in each task, respondents reveal implicit trade-offs, enabling researchers to infer preference heterogeneity (35). This study complied with the Strengthening the Reporting of Observational Studies in Epidemiology (STROBE) guidelines for cross-sectional studies and adhered to the Discrete Choice Experiment Reporting Checklist (DIRECT) for DCE reporting (36).

### 2.2 Determination of attributes and levels

A key step in implementing a DCE is to determine the attributes and their levels. It must accurately reflect the characteristics of the alternative options and ensure that respondents can understand and perceive them as reasonable (37). Firstly, we retrieved previous literature to extract the most meaningful attributes based on our assessment and the frequency of documentation. Secondly, an expert panel comprising three chief physicians of the gynecological cervical outpatient department, one public health Ph.D., and one senior laboratory technician in microbiology helped identify additional attributes. Through comprehensive evaluation and rigorous selection of all candidate attributes, the panel ultimately identified the five most critical attributes and corresponding levels. Finally, a focus group comprising 6 women aged 18 ~ 64 in Hohhot (3 from urban areas and 3 from rural areas) was recruited to optimize the expressions for attributes and levels without altering the original meaning in order to validate the understandability. The attributes and their levels are detailed in Table 1, and the detailed information on determining attributes and levels is provided in the Supplementary materials.

**Table 1:** Attributes and levels of the discrete choice experiment on HPV self-sampling.

Attributes	Levels	Operational definition and explanation
Accuracy		Concordance of the self-sampling result with a clinician-collected test.
	Accurate	The results were highly consistent.
	Moderate	The results were largely consistent, but minor discrepancies may exist.
	Inaccurate	The results showed significant differences.
Procedural difficulty		Perceived complexity for a woman to perform the self-sampling procedure herself.
	Easy	Very few steps, can be completed easily without guidance.
	Moderate	Requires careful following of instructions, but can be completed independently.
	Difficult	Complex steps, may require assistance or multiple attempts.
Comfort		Physical sensation experienced during the self-sampling process.
	Comfortable	Almost no discomfort.
	Moderate	Mild discomfort, but tolerable.
	Uncomfortable	Significant discomfort or pain.
Sampling time		Time required to collect the sample by oneself.
	<3 min	
	3 − 5 min	
	>5 min	
Price		Out-of-pocket cost for a single self-sampling.
	0 CNY	
	50 CNY	
	150 CNY	
	300 CNY	

### 2.3 Questionnaire development and experimental design

The comprehensive questionnaire comprised two components: a DCE section and a sociodemographic section. The DCE section preceded the sociodemographic section as it required greater cognitive engagement from respondents. The DCE section encompassed several choice tasks; respondents were asked to weigh and compare attributes at different levels and ultimately select their preferred HPV self-sampling scheme. The sociodemographic section captured respondents’ sociodemographic characteristics, encompassing gender, age, ethnicity, region, nature of residence, education level, career status, occupation, monthly income, and attitude toward HPV self-sampling testing.

**Figure 1:**
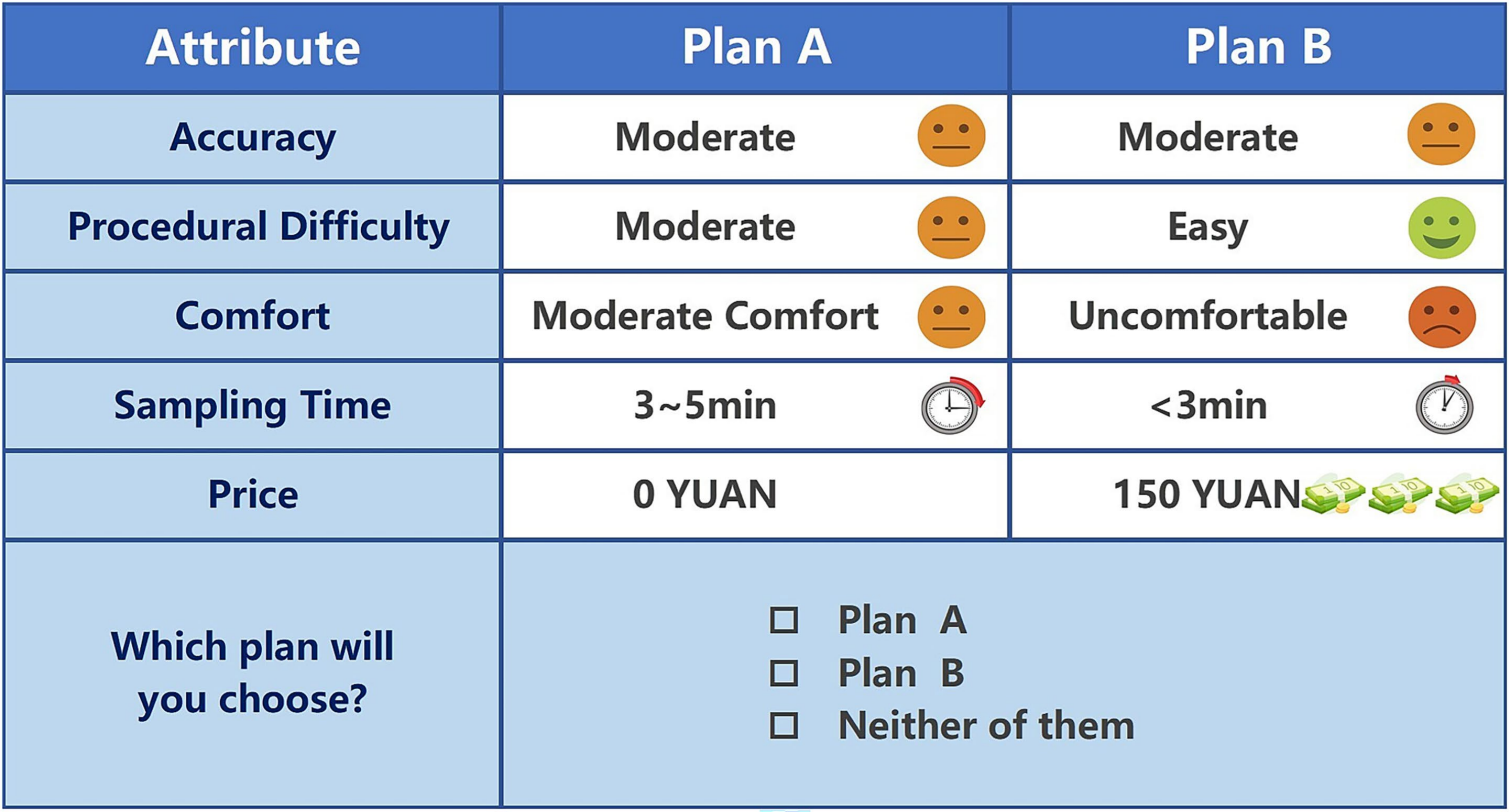
Example of a discrete choice experiment task for HPV self-sampling (English version). Survey Introduction: Cervical cancer is one of the most common malignant tumors of the female reproductive system, and persistent human papillomavirus (HPV) infection is a high-risk factor for the development of cervical cancer. Therefore, timely diagnosis is of utmost importance for eliminating persistent HPV infection and preventing its progression to cervical cancer. Considering privacy and convenience, HPV self-sampling testing may become the mainstream method in the future. Assuming you need to undergo HPV self-sampling testing, there are two self-sampling protocols available to you (Option A and Option B). Except for the listed attributes, all other attributes of Option A and Option B are identical by default. Please select the option you prefer based on your personal circumstances. If you do not prefer either option, you may choose neither.

The experimental design included five core attributes, each with three to four levels. A full factorial design would theoretically generate 324 possible profiles (3^4^ × 4^1^). To reduce respondent cognitive load and ensure experimental efficiency, an orthogonal main effects design was generated using IBM SPSS Statistics 26. This design yielded eight choice tasks, each comprising two unlabeled alternatives (described as Option A and Option B) and one exit option. The orthogonal design ensures a balanced occurrence of each level, minimizes correlation between attributes, and maximizes the accuracy of main effect estimation. To facilitate a consistency test during data quality control, one choice task was deliberately repeated within the questionnaire (38). Additionally, pictorial illustrations were employed alongside the selection sets to enhance respondents’ comprehension of the attributes and levels. The order of the choice tasks and the appearance of attributes within each task were fixed for all respondents and were not randomized. An example choice task is presented in Figure 1.

### 2.4 Sample size and inclusion/exclusion criteria

A standard computational formula exists for determining the minimum sample size in DCE, defined as follows (39). *N* represents the recommended minimum sample size, *t* represents the number of choice tasks, *a* represents the number of discrete options per choice task, and *c* represents the largest number of attribute levels across all attributes.


N≥500c/ta.

Based on this formula, we determine our minimum sample size of *N* = 125 (*t* = 8, *a* = 2, *c* = 4). It is important to clarify that the “samples” referred to here are the completed survey responses from participants, not biological specimens. The data collected were used exclusively for this research.

The inclusion criteria of this study: (1) female, aged 18 to 64, with a household registration or permanent resident of Hohhot City (this age range covers the primary target population for cervical cancer screening in China); (2) have a clear demand or willingness for cervical cancer screening; (3) be able to clearly understand the meaning expressed by each item in the questionnaire and complete it independently or with the assistance of an investigator. Exclusion criteria: (1) involuntary participation in this research; (2) patients with cognitive impairment, mental abnormalities, or critical conditions who are unable to complete the questionnaire.

### 2.5 Participant recruitment

This study adopted convenience sampling based on medical facilities and conducted recruitment of participants who met the inclusion criteria at Inner Mongolia Maternal and Child Health Hospital in Hohhot, China, which took place from September 2024 to March 2025. This site was selected as a major regional center for women’s health services, providing access to a diverse pool of potential participants from the target population.

The recruitment process was systematic and involved several steps. Trained research assistants were stationed in the hospital’s outpatient gynecology departments and general wellness check-up areas. They proactively approached women in waiting areas, briefly introduced the study’s purpose, focusing on understanding preferences for cervical cancer screening, and conducted an initial verbal screening for eligibility. For eligible women who expressed interest, the research assistants provided a detailed explanation of HPV self-sampling. They explained that the procedure involves using a sterile brush to gently collect cells from the vagina—similar to inserting a tampon—and that the sample is then sent to a laboratory for HPV DNA testing. They emphasized that the procedure is safe, generally painless, and provides results comparable to those obtained through clinician-collected sampling when using validated PCR-based assays. To aid comprehension, the research assistants showed illustrative images depicting the key steps of the procedure and, when appropriate, played a short instructional video. Additionally, recruitment posters containing a QR code linking to the online survey were displayed in prominent locations within the hospital. These posters also featured the same explanatory materials, ensuring that all potential participants had access to consistent, standardized information about HPV self-sampling prior to completing the questionnaire.

The recruitment strategy aimed to ensure wide geographic coverage and demographic relevance. We set predetermined goals for each geographical region and key age group based on local population data. Therefore, participants were sourced from across Hohhot, covering all four districts, five counties, and the economic development zone. This not only achieved extensive regional representativeness, but also their age distribution was basically consistent with that of the adult female population in this region.

### 2.6 Data collection and quality control

Data collection work was carried out from September 2024 to March 2025. Prior to participation, Eligible individuals who expressed willingness to participate were provided with an electronic informed consent form integrated into the initial part of the questionnaire on the online platform. Consent was obtained electronically before respondents could proceed to the survey. The questionnaire was designed to facilitate understanding. It incorporated pictorial aids alongside textual descriptions to illustrate the attributes and levels within the DCE choice sets. After completing the nine DCE choice sets focused on HPV self-sampling preferences, each respondent answered a short sociodemographic questionnaire. On average, participants took approximately 3.36 min to complete the entire questionnaire. As a token of appreciation for their time and to encourage future screening engagement, each participant received an HPV self-sampling kit upon full completion of the survey.

To ensure questionnaire data quality, we implemented a multi-step validation process. Questionnaires were considered invalid and subsequently excluded from the final analysis based on the following *a priori* criteria. This was assessed by including a repeated choice task within the questionnaire, if a respondent’s choices for these two identical tasks were inconsistent, the questionnaire was flagged as invalid. Questionnaires completed in less than 1 min were excluded, as this was deemed insufficient time to read and thoughtfully consider the nine DCE choice tasks. Questionnaires where the respondent selected the same option (e.g., always Option A, always Option B, or always the opt-out) across all choice tasks without any variation were excluded, as this pattern suggests a lack of engagement with the attribute trade-offs. During data screening, a dual-review system was implemented by two independent researchers to verify data integrity against these criteria.

To evaluate the feasibility, clarity, and acceptability of the questionnaire prior to its full-scale administration, a brief pilot test was incorporated at the outset of the formal data collection phase. This approach aimed to mitigate potential sample loss that could arise from conducting a separate pilot study. Specifically, the first 10 completed valid questionnaires were treated as an integrated pretest batch. These responses were reviewed for completeness, logical consistency in choices, and participants’ understanding of the task sets. The review indicated that participants were able to comprehend and complete all choice sets without notable confusion or technical issues. Based on these findings, the questionnaire was considered suitable for continued administration, and no revisions were made before proceeding with the full survey.

### 2.7 Statistical methods

Data analysis was performed using Nlogit 6 (Econometric Software, Inc., Plainview, United States). Results are presented as coefficients (*β*) with their standard errors (SE), 95% confidence intervals (CI), and *p*-values for the mean preference estimates. To quantify preference heterogeneity, the standard deviations (SD) of the random parameters and their significance levels are also reported. Willingness-to-pay (WTP) estimates were derived from the ratio of coefficients, while sociodemographic characteristics are summarized as percentages. The analysis employed a mixed logit model, which accounts for preference heterogeneity by allowing attribute parameters to follow random distributions (assumed normal) across the respondent population. This approach represents the standard analytical method for DCE data (36, 39). The utility function is defined as follows:


$$U_{\mathrm{ji}}=\beta_{0}+\beta_{1}X_{1\mathrm{ji}}+\beta_{2}X_{2\mathrm{ji}}+\ldots +\beta_{m}X_{\mathrm{mji}}+\varepsilon_{\mathrm{ji}}$$

In the model specification, econometric analysis employed dummy coding for all categorical attributes (accuracy, procedural difficulty, comfort, sampling time), with a specified reference level for each. The price attribute was modeled as a continuous variable. The attributes not assigned to the reference category were parameterized as random coefficients following normal distributions. Meanwhile, the price attribute was specified as a random parameter following a log-normal distribution to ensure all respondents have a negative price coefficient and to account for heterogeneity in price sensitivity. The alternative-specific constant (ASC), representing the opt-out option, was included as a fixed parameter. To investigate the sources of heterogeneity in the baseline preference for opting out, we interacted the ASC with respondents’ personal characteristics. The utility function *U_ji_* for individual *i* and alternative *j* is specified as a linear-in-parameters function of HPV self-sampling attributes (*X_1ji_* to *X_mji_*). *β₀* denotes the constant term, and *β_1_* to *β_m_* represent attribute coefficients estimated through the statistical modeling. These coefficients quantify the direction and magnitude of each attribute’s influence on preference degree, with *ε_ji_* being the stochastic error term. Model parameters were estimated via maximum likelihood estimation in a mixed logit model using 2,000 Halton sequence draws. Overall model significance was assessed using the log-likelihood (LL) statistic, fit was measured by McFadden’s pseudo-*R*^2^, while model selection relied on the Akaike Information Criterion (AIC) and Bayesian Information Criterion (BIC) (36).

WTP was also calculated, as the negative ratio of the coefficient for any non-price attribute to the coefficient of the price attribute. This metric quantifies the additional monetary value individuals would assign to access improved levels of a non-price attribute. The calculation formula is displayed below, where *β_x_* represents the coefficient of each attribute level, and *β_price_* represents the coefficient of the price attribute. Positive WTP values indicate the amount respondents were willing to pay *extra* to obtain a preferred attribute level (compared to the reference level), while negative values represent the compensation (price reduction) they would require to accept a less desirable level.


$$\mathrm{WTP}=-\beta_{x}/\beta_{\text{price}}$$

The relative importance (RI) of each attribute was calculated by the formula below, comparing the part-worth utility range for that attribute (defined as the difference between its highest and lowest coefficients across all levels, as Δ*β_x_*), relative to the sum of such ranges across all attributes (as ΣΔ*β_x_*). This metric quantifies each attribute’s proportional contribution to the total utility variation driving respondents’ choices.


$${\mathrm{RI}}_{x}=\frac{\Delta \beta_{x}}{\sum \Delta \beta_{x}}\times 100\%$$

Interaction analyses were performed to examine preference heterogeneity across sociodemographic subgroups. Sociodemographic characteristics were coded with different numbers and interacted with attribute levels to generate characteristic-level interaction terms, which were incorporated into the mixed logit model. This extended model augments the base specification by including product terms between level variables and sociodemographic covariates (40).

All analyses above employed a significance level of *p* = 0.05. For continuous variables, 95% confidence intervals were reported to ensure robust interpretation and policy relevance of the findings.

## 3 Results

### 3.1 Participant characteristics

A total of 246 participants completed the questionnaire between September 2024 and March 2025, of which 200 (81.3%) passed the validity test and were included in the final analysis. The study sample comprised women aged 18 to 64 years who were either registered or permanent residents of Hohhot. The average age of the respondents was 34.37 years. Among them, 29.5% were ethnic minorities, 53.5% were non-agricultural household residents, and 74.5% had higher education. The majority (65.5%) were employed, and 67% reported a monthly household income of 5,000 yuan or less. Most respondents (40.5%) preferred self-sampling for HPV when economic, time, and geographical factors permitted; 50% of respondents believe the advantage of self-sampling lies in its privacy. However, 51.5% of respondents find self-sampling challenging and worry about inaccuracies in the sampling location. Detailed sociodemographic characteristics of the participants are presented in Table 2.

**Table 2:** Sociodemographic characteristics of the study participants (*N* = 200).

Characteristics	*n* (%)
Age (years)	
18–25	66 (33.0%)
25–35	54 (27.0%)
35–45	32 (16.0%)
45–55	23 (11.5%)
55–65	25 (12.5%)
Ethnicity	
Han	141 (70.5%)
Mongolian	44 (22.0%)
Other ethnic minorities	15 (7.5%)
Household registration	
Agricultural	93 (46.5%)
Non-agricultural	107 (53.5%)
Education level	
No formal education	5 (2.5%)
Primary school	2 (1.0%)
Junior high school	10 (5.0%)
High school	8 (4.0%)
Junior college	26 (13.0%)
Undergraduate	126 (63.0%)
Master	22 (11.0%)
PhD (doctor of philosophy degree)	1 (0.5%)
Occupational status	
Employed	131 (65.5%)
Student	16 (8.0%)
Retired	12 (6.0%)
Freelancer	23 (11.5%)
Unemployed and jobless	18 (9.0%)
Monthly income per capita (CNY)	
<3,000	47 (23.5%)
3,000–5,000	87 (43.5%)
5,001–9,000	46 (23.0%)
>9,000	20 (10.0%)
HPV sampling preference	
Self-sampling	81 (40.5%)
Doctor sampling	56 (28.0%)
Mixed sampling	63 (31.5%)
Perceived advantages of self-sampling	
Low cost	19 (9.5%)
Autonomy	22 (11.0%)
Privacy	100 (50.0%)
Convenience	44 (22.0%)
Comfort	3 (1.5%)
Reducing medical resource pressure	10 (5.0%)
Others	2 (1.0%)
Concerns about self-sampling	
Difficulty (fear of incorrect sampling site)	103 (51.5%)
Unsafety (fear of physical harm)	40 (20.0%)
Inaccuracy during transportation	26 (13.0%)
Unreliable results	17 (8.5%)
Timeliness of feedback	7 (3.5%)
Others	7 (3.5%)

### 3.2 Preferences for HPV self-sampling attributes

**Figure 2:**
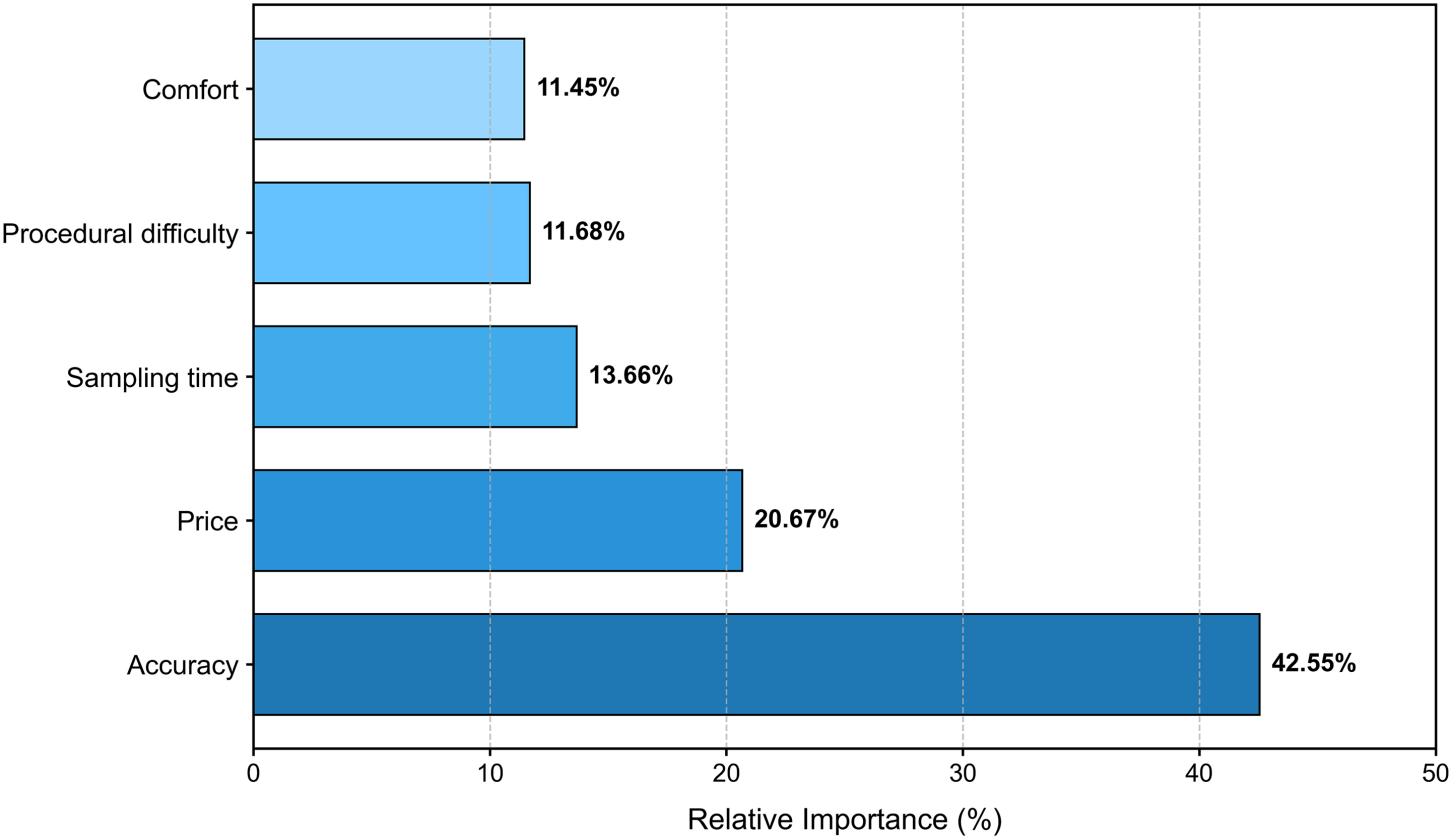
Relative importance of attributes in women’s preferences for HPV self-sampling.

**Figure 3:**
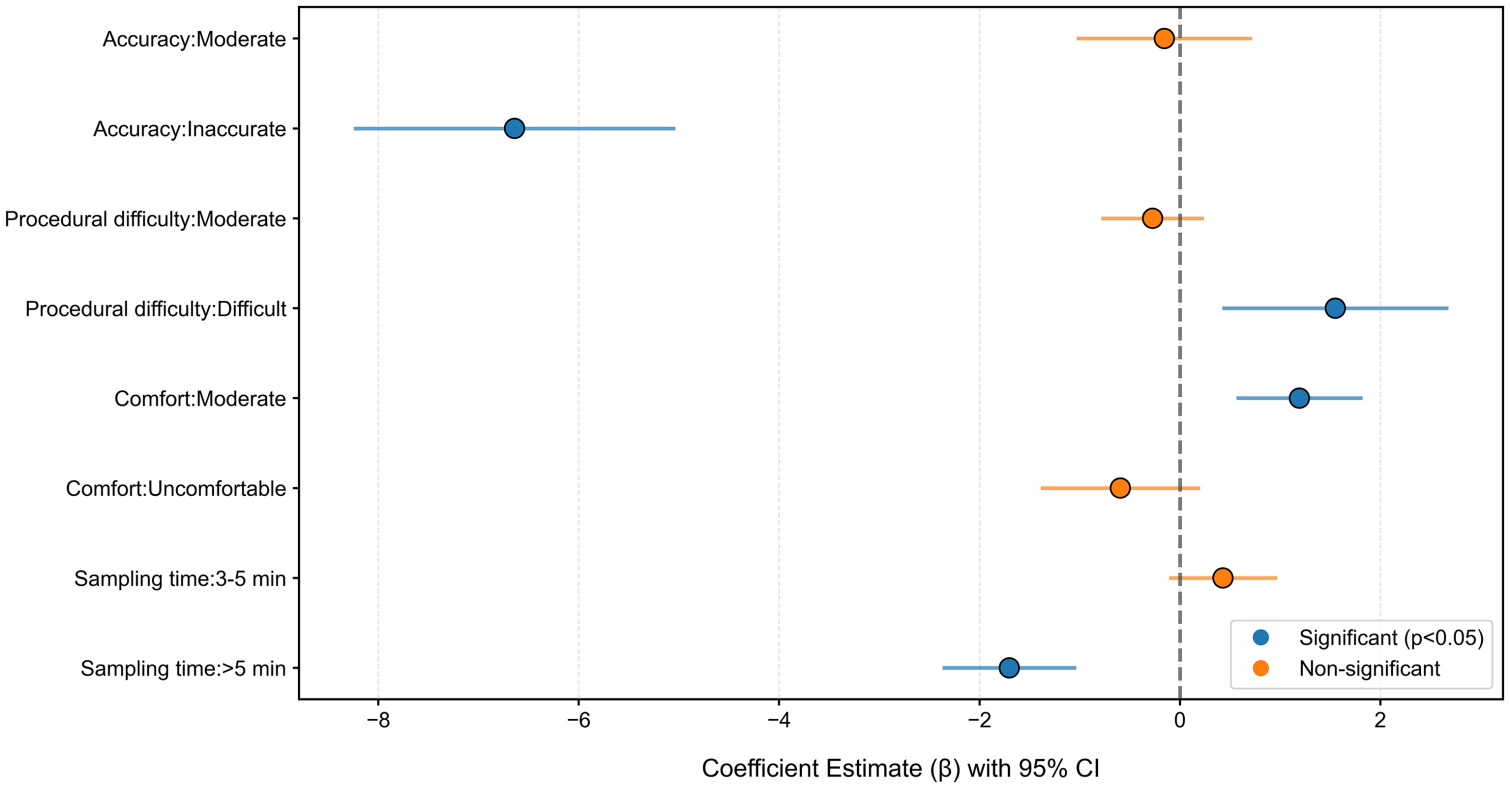
Preference for HPV self-sampling attributes from the mixed logit model.

The mixed logit model exhibited excellent fit, with a McFadden’s pseudo-*R*^2^ of 0.49. The final model statistics were: LL = −895.57, AIC = 1,829.1, BIC = 1,931.3. Figure 2 illustrates the emphasis placed by women in Hohhot on the attributes of HPV self-sampling, ranked as follows: accuracy (42.55%) is the highest, followed by price (20.67%), time required for sampling (13.66%), procedural difficulty (11.68%), and comfort level (11.45%). As shown in Figure 3 and Table 3, the target group strongly disfavored inaccurate HPV self-sampling tests (*β* = −6.640, *p* < 0.001). The price coefficient was significantly negative (*β* = −0.011, *p* < 0.001), indicating that higher costs reduce the utility of the self-sampling option. The marginal rate of substitution between price and other attributes is reflected in the WTP estimates. When the self-sampling operation time exceeded 5 min, it led to reduced acceptability (*β* = −1.703, *p* < 0.001). Notably, Respondents exhibited a positive preference for more difficult procedures of HPV self-sampling (*β* = 1.550, *p* < 0.01). This phenomenon warrants further investigation into the influence of medical device design characteristics and user perceptions. A moderate level of procedural comfort was also associated with higher acceptance (*β* = 1.192, *p* < 0.001).

**Table 3:** Results of the mixed logit model analyzing preferences for HPV self-sampling attributes.

Attributes and levels	*β*	SE	*P*	95% CI		SD	*P-* (SD)	WTP (CNY)
				Inf.	Sup.			
Accuracy (ref: accurate)								
Moderate	−0.156	0.447	0.727	−1.032	0.719	2.668	<0.001	−14.49
Inaccurate	−6.640	0.819	<0.001	−8.245	−5.036	5.363	<0.001	−617.13
Procedural difficulty (ref: easy)								
Moderate	−0.273	0.262	0.296	−0.786	0.240	1.275	<0.001	−25.41
Difficult	1.550	0.575	0.007	0.422	2.678	4.405	<0.001	144.08
Comfort (ref: comfortable)								
Moderate	1.192	0.320	<0.001	0.564	1.820	0.619	0.213	110.82
Uncomfortable	−0.595	0.405	0.142	−1.389	0.200	1.421	<0.001	−55.28
Sampling time (ref: <3 min)								
3–5 min	0.429	0.275	0.119	−0.110	0.969	0.941	0.010	39.91
>5 min	−1.703	0.340	<0.001	−2.370	−1.036	0.840	0.028	−158.33
Price (CNY)	−0.011	0.001	<0.001	−0.014	−0.008	0.002	0.553	
ASC	4.913	0.415	<0.001	4.100	5.725			

ASC, alternative specific constant; SE, standard error; CI, confidence interval; SD, standard deviation; WTP, willingness-to-pay; WTP derived from unrounded coefficients; reported *β* values are rounded to 3 decimals, so minor discrepancies may appear upon recalculation; mixed logit model with 2000 Halton draws; random parameters (normally distributed) were specified for all attributes except the ASC; *β*, mean preference coefficient. *p*, p-value for the test of β ≠ 0; SD, estimated standard deviation of the random coefficient, indicating preference heterogeneity. *p* (SD): *p*-value for the test of SD > 0 (significant value confirms heterogeneity); Dummy coding was used for attributes; reference levels are as indicated in the table.

### 3.3 Willingness to pay (WTP)

The results, presented in Table 3, reveal substantial and statistically significant monetary valuations associated with key attributes. Accuracy was the most valued attribute. Respondents demanded a significant price reduction of 617.13 CNY to accept an Inaccurate test compared to an Accurate one, underscoring its non-negotiable status. Operational Characteristics showed clear monetary thresholds. Notably, respondents were willing to pay a premium of 144.08 CNY for a device perceived as Difficult to use over one that was Easy, a counterintuitive finding that may relate to perceptions of procedural rigor. For comfort, they valued a moderate comfort level, with a WTP of 110.82 CNY over the reference (Comfortable). Regarding sampling time, a procedure taking >5 min necessitated a significant price cut of 158.33 CNY.

### 3.4 Heterogeneity analysis of respondents’ preferences for HPV self-sampling

As shown in Table 4, all interaction terms have been tested at a significance level of 5%, while other interaction terms that did not reach significance have not been displayed, indicating the robustness of the model’s estimated preference heterogeneity with respect to key sociodemographic factors. Preference heterogeneity was observed in relation to age. Acceptance of inaccurate results (*β* = 0.190, *p* < 0.001), preference for procedurally difficult self-sampling (*β* = 0.106, *p* < 0.01), and preference for moderate comfort (*β* = 0.037, *p* < 0.05) all increased with age. Compared with Han respondents, ethnic minorities showed a weaker aversion to inaccurate results (*β* = 3.085, *p* < 0.01). In contrast, a higher education level was associated with a lower preference for moderate comfort (*β* = −0.375, *p* < 0.05). Moreover, women who preferred self-sampling over clinician sampling demonstrated a particularly aversion to low accuracy (*β* = −2.623, *p* < 0.001) and procedural difficulty (*β* = −1.095, *p* < 0.05).

**Table 4:** Interaction effects between sociodemographic characteristics and preferences for attributes of HPV self-sampling.

Attributes/characteristics	*β*	SE	*p*	95% CI	
				Inf.	Sup.
Age × inaccurate	0.190	0.038	<0.001	0.114	0.265
Age × difficult	0.106	0.035	0.002	0.038	0.173
Age × moderate comfort	0.037	0.015	0.011	0.009	0.066
Age × ASC	0.211	0.054	<0.001	0.106	0.316
Ethnicity × inaccurate	3.085	1.092	0.005	0.945	5.225
Education level × moderate comfort	−0.375	0.176	0.033	−0.719	−0.030
Sampling preference × inaccurate	−2.623	0.685	<0.001	−3.965	−1.280
Sampling preference × difficult	−1.095	0.483	<0.023	−2.041	−0.150

ASC, alternative specific constant; SE, standard error; CI, confidence interval. Age (continuous): positive coefficient indicates stronger preference (or weaker aversion) with increasing age. Ethnicity (dummy, reference = Han): coefficient reflects the difference relative to Han ethnicity. Education (ordinal, from primary to postgraduate): coefficient is relative to the lowest education category. Sampling preference (categorical, reference = clinician sampling): coefficients are interpreted relative to those preferring clinician sampling.

## 4 Discussion

### 4.1 Principal findings

This DCE assessed preferences for HPV self-sampling attributes among women in Hohhot, China. The study estimated relative importance (RI), quantified preferences, derived willingness-to-pay (WTP), and explored heterogeneity through interaction terms. The analysis identified test accuracy as the primary driver of preference, accounting for the largest share of RI at 42.55%, followed by out-of-pocket cost at 20.67% and self-collection time at 13.66%. Perceived procedural complexity (11.68%) and physical comfort (11.45%) were comparatively less influential. The mixed logit model confirmed that low accuracy, extended sampling times (>5 min), and higher costs significantly diminished choice probability. Notably, the analysis revealed a significant positive preference for procedures described as operationally difficult, alongside a tendency to favor moderate comfort over extremes. Moreover, significant preference heterogeneity was observed across variables such as age, ethnicity, education level, and HPV sampling preference. These findings illuminate the fundamental needs and trade-off considerations of women, offering valuable insights for enhancing regional cervical cancer screening strategies.

### 4.2 Information intervention and preference stability

A legitimate concern in any DCE involving a novel health technology is whether respondents possess sufficient understanding to make valid trade-offs. In this study, we addressed this concern through a two-tiered information strategy. First, during recruitment, trained research assistants provided a detailed, standardized explanation of HPV self-sampling. This approach ensured that all participants, regardless of prior awareness, acquired a foundational understanding of the procedure before entering the survey. Second, within the DCE itself, each choice task was accompanied by pictorial illustrations clarifying the meaning of each attribute level. This combination of pre-survey education and in-task visual aids equipped participants to construct a coherent cognitive framework for evaluating the hypothetical scenarios.

DCEs are inherently designed to elicit preferences based on clearly described attributes rather than relying on respondents’ pre-existing expertise (31, 32). As Szinay et al. noted, for innovative digital health products, well-constructed DCEs can enable respondents to form reasoned preferences even without prior experience, provided the information presented is comprehensible and trade-offs are clearly framed (31). In our study, the integration of pre-task education and in-task visual clarification served as an “instant educational intervention,” allowing participants to make informed choices that reflect their underlying values rather than superficial impressions. The robustness of our DCE results further supports this interpretation, the mixed logit model demonstrated excellent fit. This study adhered to the DIRECT, ensuring methodological transparency and alignment with established best practices for preference elicitation (36). Moreover, the significant standard deviations observed for most random parameters indicate that the model successfully captured meaningful preference heterogeneity rather than producing spurious estimates due to respondent confusion. These metrics collectively attest to the internal validity of the experiment and the reliability of the elicited preferences.

Nevertheless, it would be overly optimistic to claim that information empowerment entirely eliminates cognitive heterogeneity. While our information strategy mitigated many knowledge gaps, it could not entirely eliminate the influence of pre-existing schemas. Future research should consider measuring health literacy or prior knowledge as covariates and testing how different intensities of information intervention affect preference stability and predictive validity. With this methodological reassurance, we now turn to comparing our findings with prior studies on HPV self-sampling, which further contextualizes the observed preference patterns within the broader literature.

### 4.3 Comparison with prior work and novel insights

Our analysis revealed that participants consistently identified test accuracy as the most important factor, underscoring their strong prioritization of reliable results. This finding is consistent with a Chinese HPV self-sampling study (41). A study in the Netherlands also showed that accuracy is the primary condition for user acceptance (42). This universal emphasis underscores that trust in the reliability of the result is a non-negotiable foundation for any self-sampling strategy.

Respondents’ ability to afford self-sampling services significantly influenced their preference decisions (19, 43), which is highly consistent with the price sensitivity observed in this study. A research paper on cervical cancer self-sampling screening in Nepal noted that cost is a key barrier to the promotion of self-sampling, particularly the pricing of kits and out-of-pocket expenses, but evidence suggests that its long-term cost-effectiveness is superior to traditional methods (44). Recommendations include reducing costs through pharmacy distribution, policy subsidies, and public education, while optimizing logistics to minimize hidden expenses.

Our findings indicate that a shorter self-sampling duration is a significant preference among women, reflecting considerations that extend beyond mere convenience. From both clinical and experiential perspectives, the length of the self-sampling procedure is intrinsically linked to the user’s physical and psychological comfort. Clinical experience suggests that prolonged sampling contact time can heighten the risk of local irritation and minor bleeding. Moreover, for many women—particularly those who are under-screened and may experience anxiety, embarrassment, or discomfort with gynecological procedures—a lengthy self-sampling process can exacerbate psychological distress. Consequently, a preference for a swift sampling process is not only rational but also indicative of a desire for procedures perceived as safer, less invasive, and more respectful of individual bodily autonomy and emotional well-being. This highlights that the efficiency of the self-sampling constitutes a critical design feature. Therefore, this study posits that the development of self-sampling should focus on optimizing this attribute. By ensuring that samples are comprehensively collected, rapid sampling can be achieved, thereby enhancing clinical safety and overall user experience. These improvements are likely to increase the adoption rate among sensitive or concerned populations.

It is noteworthy that, while HPV self-sampling is generally designed to be a straightforward procedure, our findings indicate that some respondents expressed a positive preference for procedures described as having greater operational complexity. This should not be interpreted as an inherent desire for complexity, but rather as a potential reflection of the cognitive associations that users may form between procedural complexity and perceived clinical rigor or reliability. However, this does not imply that self-sampling devices should be deliberately made complex or cumbersome. This underscores that user perception, rather than objective ease, often drives acceptance. Importantly, such perceptions can be positively reshaped through clear guidance. For example, one study demonstrated that video-assisted instructions significantly improved perceived ease and future willingness to self-sample among participants (45). This highlights that well-designed informational support can mitigate anxiety linked to perceived complexity without altering the procedure’s inherent simplicity. Thus, in promoting self-sampling, the focus should be on ensuring that instructions and design minimize ambiguity and reinforce accessibility—actively managing user perception to build trust while preserving actual ease of use.

The comfort advantages of self-sampling devices can be translated into improved actual coverage rates that have been established as a key factor influencing women’s screening preferences (46). In a rural Zimbabwean (19), women demonstrated the strongest preference for the comfort of sampling devices (OR = 0.312), far exceeding other attributes. Similarly, a study in South Africa also mentioned that self-sampling is comfortable and painless, with 88.4% of women preferring self-sampling (47). When comfort reaches an acceptable level (e.g., no significant pain or bleeding), further optimization has a diminishing marginal effect on decision-making. It better meets their need for moderate comfort rather than pursuing higher comfort levels. This phenomenon may be related to women’s experiences with healthcare services in Inner Mongolia. Previous studies have shown that women in areas with scarce healthcare resources have a higher tolerance threshold for medical discomfort (48).

Based on the WTP analysis, respondents were willing to pay a premium of up to 617.13 CNY to avoid an inaccurate test, far exceeding the upper limit of the experimental price range (300 CNY). This reaffirms that accuracy is a non-negotiable core attribute in decision-making. In contrast, the compensation required for shortening sampling time (>5 min), while significant (158.3 CNY), was substantially lower than the valuation placed on accuracy. This indicates that, provided accuracy is assured, optimizing the operational process and avoiding excessively long duration is key to improving acceptability. The price coefficient (*β*) was significantly negative, but its standard deviation (SD) was not statistically significant (*P_SD_* = 0.553), suggesting that no significant unobserved heterogeneity in price sensitivity was detected among respondents within this model specification. This implies that, in general, most individuals shared a similar preference for lower prices. Therefore, the absence of significant heterogeneity in the price parameter indicates that a uniform and cost-effective pricing strategy may be broadly applicable to the majority. Strategies should instead focus on ensuring affordability and accessibility at a competitive price point, rather than relying on complex tiered pricing based solely on varying levels of price sensitivity (21).

Given the significant price sensitivity observed in our study (*β* = −0.011, *p* < 0.001) and the fact that 67% of respondents reported a monthly income below 5,000 CNY, addressing financial barriers is paramount. While women place a high monetary value on accuracy, this reflects a theoretical valuation, not an ability to pay out-of-pocket. Therefore, implementation models must actively reduce upfront costs. Our findings strongly support integrating HPV self-sampling into existing public health programs with government subsidies to make it affordable for all, especially low-income groups. Furthermore, innovative community-based financing models, such as the ‘pay-it-forward’ approach currently being piloted in Hohhot (28), hold great promise. In this model, a person receives a subsidized or free kit and is later given the option to donate to fund screening for someone else. This approach leverages community solidarity to sustain the program while directly mitigating the financial barrier highlighted by our DCE, offering a culturally appropriate and economically viable pathway to scale up coverage in underserved regions.

This study’s methodology for examining preference heterogeneity, which includes the incorporation of interaction terms between respondent characteristics and attributes, is informed by the ISPOR Good Research Practices for Conjoint Analysis Task Force. This guide advocates for the investigation of preference heterogeneity through the development of interaction terms that link individual characteristics with specific attributes (49). This study confirmed that there was a significant heterogeneous preference for HPV self-sampling among the interviewed women with different demographic characteristics, offering valuable insights for the development of targeted public health strategies.

Age significantly influenced preferences for multiple attributes. While the overall preference heterogeneity for moderate comfort was limited (SD = 0.619, *p*_SD_ = 0.213), its interaction with age was observed, indicating that age systematically moderates the valuation of both the direction and intensity of preference for moderate comfort. Older women were more tolerant of lower accuracy and showed greater acceptance of moderate comfort. Preferences across age groups are not opposites but represent different weighting of similar attributes. Both groups value accuracy and comfort, but young women place a higher premium on these as preconditions for participation, while older women may weigh them against prior screening habits. Future interventions should move beyond uniform messaging by tailoring communications to different age groups. For instance, highlighting precision and efficiency for younger women, while underscoring reliability, support availability, and procedural clarity for older women. All messaging should maintain a consistent, evidence-based foundation regarding the test itself. As women age, their preference for difficult operations increases, which may stem from their association of complex operations with professionalism. This highlights uncontrolled confounding factors in the experiment, as previously discussed, such as prior familiarity with sampling techniques and differences in acceptance of new technologies. Future studies should incorporate these variables to clarify the underlying causes.

The ASC interaction term is used to capture unobserved attributes or inherent preferences for options, directly reflecting the inherent bias of individual characteristics toward “selecting a particular type of scheme”. It quantifies the moderating effect of demographic variables on baseline participation probability. This study shows that older age groups are more likely to choose the exit option, which reflects a different type of engagement that could still be fragile if the self-sampling option does not meet their expectations for accuracy, simplicity, and convenience. Future research should explore the qualitative drivers behind the exit tendency in older adults and the specific barriers for younger women to better tailor support tools and communication frameworks across the age continuum.

Additionally, we found that ethnicity moderated the aversion to inaccuracy, indicating a weaker aversion among ethnic minority groups compared to the Han majority. This finding aligns with prior research indicating that ethnic minority women in China possess lower levels of knowledge and hold more negative attitudes toward cervical cancer screening compared to their Han counterparts (50, 51). For example, Wu et al. (2018) reported that among female college students from seven ethnic groups, minority students showed significantly lower acceptance of screening compared to Han students (50). Similarly, a study on ethnic minority women in Yunnan found that although they initially had skeptical attitudes, these women showed high acceptance of self-sampling cervical cancer screening after receiving appropriate education (27). In summary, these findings suggest that the weaker aversion to inaccurate information observed in the ethnic minority population may stem from the inability to access health information that is in line with their culture and language, rather than a lack of concern for health outcomes. Therefore, through targeted and culturally sensitive education to enhance knowledge levels, not only can the screening participation of these populations be improved, but they can also have a more accurate understanding of risks and make more informed decisions.

We also found that higher education levels were associated with a reduced preference for moderate comfort, indicating that respondents with higher educational attainment exhibit a more pronounced differentiation between comfort levels. Furthermore, women who demonstrated a pre-existing preference for self-sampling displayed a markedly stronger aversion to difficult procedures as well as to low accuracy. This suggests that individuals most inclined toward self-sampling prioritize ease of use and reliability. For this group, perceived difficulty is not regarded as an indicator of quality but rather as a practical obstacle. Their rejection of inaccuracies indicates that the accuracy of test results is a core consideration and foundational trust threshold when choosing self-sampling methods.

### 4.4 Recommendations for implementation

Based on our findings, we propose an integrated framework to enhance the uptake of HPV self-sampling. First, establishing trust and ensuring accessibility are foundational. Promotional efforts must prioritize communicating the high diagnostic accuracy of self-sampling to mitigate skepticism. Simultaneously, economic and physical barriers should be reduced through subsidies, pharmacy distribution, or inclusion in public health insurance schemes.

Second, the user experience should be optimized for simplicity and reassurance. Self-sampling kits must be intuitive, with instructions designed to lower the perceived technical threshold. For instance, including QR codes on kits that link to concise instructional videos can serve a dual purpose: they demystify the procedure to reduce anxiety and reinforce the test’s medical rigor. This ensures that a “simple” process is still perceived as professional and high-quality care.

Third, communication strategies must be tailored to the diverse demographic profile of the region. A segmented approach is recommended: messaging for younger women might emphasize privacy, efficiency, and accuracy, whereas outreach to older or less literate groups should focus on reliability and step-by-step support. Crucially, in ethnically diverse regions like Inner Mongolia, cultural and linguistic adaptation is non-negotiable. Materials should be available in local ethnic languages and disseminated through culturally familiar channels—such as community gatherings or oral explanations—to bridge the gap between Han and minority populations.

Finally, sustainable implementation requires systemic integration. Self-sampling should be embedded into routine primary healthcare and community services, leveraging endorsements from healthcare providers to legitimize it as a standard screening option. Future research should extend beyond urban centers to remote pastoral areas to validate these preferences across the broader population.

### 4.5 Limitations

This study has limitations. First, although our sample size met the minimum requirement, it was relatively modest for detecting complex interaction effects, particularly in subgroup analyses. Additionally, our reliance on convenience sampling resulted in an overrepresentation of urban, highly educated women, whose preferences likely differ from those in remote agricultural or traditional pastoral areas. Consequently, the observed ethnic heterogeneity primarily reflects the views of urbanized minorities and may not fully capture the distinct cultural or logistical constraints faced by women in nomadic communities. Future research with larger and more diverse samples is warranted to validate and extend our findings, with priority given to recruiting participants from these underrepresented regions.

Second, the attribute set and levels—although evidence-informed—cannot capture all determinants of HPV self-sampling. While we utilized qualitative labels to accommodate varying health literacy, the subjective interpretation of these terms could affect the precision of preference estimates. Future studies might incorporate quantitative metrics (e.g., sensitivity/specificity percentage) to enhance clarity and policy relevance. Additionally, the survey was administered exclusively in Mandarin. Although visual aids and detailed explanations were provided to standardize comprehension, the absence of a Mongolian version and variations in participants’ prior knowledge of self-sampling may have introduced subtle misunderstandings or influenced perceptions of procedural difficulty, particularly among minority participants.

Finally, as with all DCEs, our results reflect stated rather than revealed preferences. We did not track the actual utilization of the self-sampling kits provided. While stated preferences offer valuable insights into decision-making trade-offs, they do not guarantee real-world behavior. To enhance predictive validity, future studies should link DCE data with actual uptake rates—for instance, by using trackable kits—to assess the concordance between women’s choices in hypothetical scenarios and their clinical screening behaviors.

## 5 Conclusion

Our study identified test accuracy as the primary determinant of HPV self-sampling preferences, followed by out-of-pocket cost and sampling time, with procedural characteristics being secondary. The significant preference heterogeneity across demographic groups suggests that effective implementation requires both optimized core attributes and tailored strategies for different user segments. The methodology and analytical framework demonstrated here offer transferable value for designing cervical cancer screening programs in similar resource-constrained settings. By quantifying preferences and translating them into stratified implementation approaches, this study provides a replicable model for aligning preventive health technologies with diverse population needs, ultimately supporting more equitable and effective cancer prevention coverage.

## Data Availability

The original contributions presented in the study are included in the article/Supplementary material, further inquiries can be directed to the corresponding author.
