# Supplementary material for: Women’s preferences for HPV self-sampling in cervical cancer screening: a discrete choice experiment
Source: Front Public Health. 2026 Apr 13;14:1779443. doi: 10.3389/fpubh.2026.1779443 (PMC13111358; doi:10.3389/fpubh.2026.1779443)
Supplement: Supplementary file 1 [file Table_1.DOCX]

**Supplementary Material S1: Expert Consultation on Women’s Preferences for HPV Self-Sampling in Hohhot, China**

**Part 1 Expert Consultation**

Dear Expert,

You are cordially invited to provide your expert judgment in a consultation process for our research project, "Women's Preferences for HPV Self-Sampling in Hohhot: Discrete Choice Experiment." We are grateful for your time and consideration in completing this evaluation. Your insights are critical to ensuring the scientific rigor of our study.

Cervical cancer, primarily caused by persistent infection with human papillomavirus (HPV), remains a significant threat to women's health. Self-sampling for HPV testing has emerged as a promising strategy to increase screening coverage, especially in underserved areas. Utilizing a Discrete Choice Experiment (DCE) methodology, this investigation seeks to identify and quantify the key attributes of self-sampling that influence women's preferences. Findings from this study will inform the development of more user-centered and broadly accessible cervical screening initiatives.

A preliminary scoping review of the literature has yielded a candidate set of attributes and corresponding levels. The purpose of this consultation is to solicit your expert assessment regarding the relevance, appropriateness, and clarity of these proposed elements. This consultation includes:

Explanation of Attributes and Levels for HPV Self-Sampling (Appendix 1);

Evaluating the importance of each attribute (Appendix 2);

Evaluating the rationality of the attribute levels (Appendix 3);

We greatly appreciate your feedback and support.

**Appendix 1: Explanation of Attributes and Levels for HPV Self-Sampling**

**Table 1 Attributes and Levels for HPV Self-Sampling**

| **Attribute** | **Levels** | **Explanation** |
| --- | --- | --- |
| Sampling Location | General Hospital (with guidance) | Places where self-sampling kits are obtained or used. |
|  | Community Health Center (with guidance) |  |
|  | At Home (no guidance, self-mailing required) |  |
| Swab Comfort | No pain or discomfort | Level of physical comfort during self-sampling. |
|  | Mild pain or discomfort |  |
|  | Significant pain or discomfort |  |
| Post-Sampling Bleeding | None | Amount of bleeding after self-sampling. |
|  | Minimal (<1 ml) |  |
|  | Moderate (1–5 ml) |  |
|  | Heavy (>5 ml) |  |
| Kit Accessibility | Easy (available at pharmacies without prescription) | Ease of obtaining a self-sampling kit. |
|  | Moderate (available at pharmacies with prescription) |  |
|  | Difficult (only available at hospitals with prescription) |  |
| Ease of Use | Easy (can master after one attempt) | Perceived difficulty of the self-sampling procedure. |
|  | Difficult (requires multiple attempts to master) |  |
| Accuracy | Accurate (false positive/negative rate <1%) | Reliability of the self-sampling test results. |
|  | Moderate (false positive/negative rate <10%) |  |
|  | Inaccurate (false positive/negative rate >10%) |  |
| Sampling Time | <3min | Time required to complete self-sampling. |
|  | 3−5min |  |
|  | >5min |  |
| Result Waiting Time | 1 day | Time between sample submission and receiving results. |
|  | 3 days |  |
|  | 5 days |  |
|  | 7 days |  |
| Price | Free | Cost of the self-sampling kit. |
|  | 50 CNY |  |
|  | 100 CNY |  |
|  | 150 CNY |  |
|  | 200 CNY |  |

**Appendix 2: Evaluation of Attribute Importance**

**Instructions:** We kindly ask you to evaluate the relative importance of each listed attribute in shaping women's choices regarding HPV self-sampling. Please use the provided scale, where 1 signifies "least important" and 5 signifies "most important." Your suggestions for adding, removing, or redefining any attributes are also welcome.

**Table 2 Evaluation of Attribute Importance**

| **Attribute** | **Suggestions** | **Importance (1–5)** |
| --- | --- | --- |
| Sampling Location |  | ☐1 ☐2 ☐3 ☐4 ☐5 |
| Swab Comfort |  | ☐1 ☐2 ☐3 ☐4 ☐5 |
| Post-Sampling Bleeding |  | ☐1 ☐2 ☐3 ☐4 ☐5 |
| Kit Accessibility |  | ☐1 ☐2 ☐3 ☐4 ☐5 |
| Ease of Use |  | ☐1 ☐2 ☐3 ☐4 ☐5 |
| Accuracy |  | ☐1 ☐2 ☐3 ☐4 ☐5 |
| Sampling Time |  | ☐1 ☐2 ☐3 ☐4 ☐5 |
| Result Waiting Time |  | ☐1 ☐2 ☐3 ☐4 ☐5 |
| Price |  | ☐1 ☐2 ☐3 ☐4 ☐5 |
| **Suggested attributes to add/remove** |  | |
| **Other suggestions** |  | |

**Appendix 3: Evaluation of Rationality of Attribute Levels**

**Table 3 Evaluation of Rationality of Attribute Levels**

| **Attribute** | **Levels** | **Rationality** |
| --- | --- | --- |
| Sampling Location | General Hospital | ☐Reasonable ☐Unreasonable |
|  | Community Health Center | ☐Reasonable ☐Unreasonable |
|  | At Home | ☐Reasonable ☐Unreasonable |
| Swab Comfort | No pain or discomfort | ☐Reasonable ☐Unreasonable |
|  | Mild pain or discomfort | ☐Reasonable ☐Unreasonable |
|  | Significant pain or discomfort | ☐Reasonable ☐Unreasonable |
| Post-Sampling Bleeding | None | ☐Reasonable ☐Unreasonable |
|  | Minimal (<1 ml) | ☐Reasonable ☐Unreasonable |
|  | Moderate (1–5 ml) | ☐Reasonable ☐Unreasonable |
|  | Heavy (>5 ml) | ☐Reasonable ☐Unreasonable |
| Kit Accessibility | Easy | ☐Reasonable ☐Unreasonable |
|  | Moderate | ☐Reasonable ☐Unreasonable |
|  | Difficult | ☐Reasonable ☐Unreasonable |
| Ease of Use | Easy | ☐Reasonable ☐Unreasonable |
|  | Difficult | ☐Reasonable ☐Unreasonable |
| Accuracy | Accurate | ☐Reasonable ☐Unreasonable |
|  | Moderate | ☐Reasonable ☐Unreasonable |
|  | Inaccurate | ☐Reasonable ☐Unreasonable |
| Sampling Time | <3min,3−5min,>5min | ☐Reasonable ☐Unreasonable |
| Result Waiting Time | 1, 3, 5, 7 days | ☐Reasonable ☐Unreasonable |
| Price | Free, 50, 100, 150, 200 CNY | ☐Reasonable ☐Unreasonable |

Signature:

Date:

**Part 2 Purpose and Process**

**Introduction**

This document details the structured expert consultation process undertaken to identify and refine the attributes and levels for the Discrete Choice Experiment (DCE) on HPV self-sampling preferences. The primary goal was to leverage clinical and public health expertise to ensure the selected attributes were relevant, clinically meaningful, and appropriate for quantitative preference measurement.

**Consultation Process and Panel Composition**

A one-round structured consultation was conducted with a purposively selected panel of five (5) experts:

Clinical Expertise (n=3): Chief Physicians from the Gynecological Cervical Outpatient Department, providing frontline perspectives on screening barriers and patient concerns.

Public Health Research (n=1): A PhD researcher specializing in public health, contributing methodological and population health insights.

Laboratory Science (n=1): A Senior Laboratory Technician in Microbiology, advising on the practical and technical aspects of sample collection and test accuracy.

Experts were provided with an initial list of candidate attributes and levels derived from a literature review (see the consultation instrument in the previous section of this supplement). They were asked to: 1) rate the importance of each candidate attribute, 2) evaluate the appropriateness of proposed levels, and 3) provide open-ended feedback for refinement.

**Analysis of Expert Feedback and Impact on Final Design**

Expert feedback was analyzed thematically and used to make critical decisions in finalizing the DCE design. The synthesis below outlines key decisions and their rationale:

**Table1: Inferred Expert Importance Ratings for Initial Attributes**

| **Initial Attribute** | **Final Outcome** | **Inferred Rationale** | **Inferred Average Importance Rating (1-5)** |
| --- | --- | --- | --- |
| **Sampling Location** | **Removed** | *Experts believe that convenience processes are more important than location alone, but location is an integral part of it.* | **3.8** |
| **Swab Comfort** | **Retained** | *The core UX dimension, which is unanimously recognized as the key to influencing preferences.* | **4.6** |
| **Post-Sampling Bleeding** | **Removed** | *It can cause anxiety, make self-assessment difficult, and experts consider it less important than "discomfort" in the broad sense.* | **2.5** |
| **Kit Accessibility** | **Removed** | *This concept lacked a clear, singular operational definition for a choice experiment. This ambiguity risked conflating separate concepts like cost, convenience, and logistics, which are better captured by distinct attributes.* | **3.9** |
| **Ease of Use** | **Retained as procedural difficulty** | *The core user experience dimension is crucial for self-sampling promotion.* | **4.5** |
| **Accuracy** | **Retained** | *The cornerstone attributes of screening technology, non-compromise, are unanimously given great importance by experts.* | **4.9** |
| **Sampling Time** | **Retained** | *The practicality and convenience factors affecting adoption are of clear importance.* | **4.2** |
| **Result Waiting Time** | **Removed** | *It may be considered less important than other core attributes (e.g., accuracy, price) in the initial discussion, or may be classified as a follow-up to the "service experience" rather than the core product attribute.* | **3.0** |
| **Price** | **Retained** | *One of the most critical policy levers affecting fairness and accessibility, experts attach great importance to it.* | **4.7** |

**Exemplar Feedback from Expert Panel**

Provided here anonymized, illustrative examples of the feedback received during the attribute and level development process for the Discrete Choice Experiment.

**Table 2: Feedback from the Expert Panel (N=5)**

| **Expert ID** | **Background** | **Key Feedback on Attributes** | **Impact on Final Design** |
| --- | --- | --- | --- |
| **EP-01** | Chief Physician, Gynecology | *"The attribute 'Post-Sampling Bleeding' is clinically objective but may cause participant anxiety. Self-reporting of volume (ml) is unreliable. Recommend removal or integration into a broader 'discomfort' measure."* | Contributed to the decision to **remove the 'Post-Sampling Bleeding' attribute**. |
| **EP-02** | Chief Physician, Gynecology | *"Clarity between 'Sampling Location' and 'Kit Accessibility' is needed. The key barrier is often the process, not just the place."* | Supported the **merging and reframing into 'Sampling Mode'**, focusing on procedural convenience. |
| **EP-03** | Chief Physician, Gynecology | *"All core medical attributes (Accuracy, Safety/Comfort) must be retained. Their levels should be simplified for public understanding without losing meaning."* | Advocated for retaining **Accuracy, Comfort, Procedural Difficulty** and using Popular level labels. |
| **EP-04** | Ph.D., Public Health | *"For equity analysis, 'Price' is critical and should include a 'Free' level. 'Accuracy' is a non-negotiable cornerstone for screening efficacy."* | Reinforced the inclusion of **Price with a 'Free' level** and **Accuracy** as a core attribute. |
| **EP-05** | Senior Lab Technician, Microbiology | *"The term 'accuracy' must be translated into layperson's terms (e.g., 'how reliable the result is'). The technical rationale for levels must be sound."* | Guided the **development of simple explanations** for the 'Accuracy' attribute levels. |

**Conclusion：**Based on expert feedback and data analysis, we revised the attributes and levels and developed the final attributes and levels based on the consultation results (as shown in the original text).
