# Supplementary material for: Women’s preferences for HPV self-sampling in cervical cancer screening: a discrete choice experiment
Source: Front Public Health. 2026 Apr 13;14:1779443. doi: 10.3389/fpubh.2026.1779443 (PMC13111358; doi:10.3389/fpubh.2026.1779443)
Supplement: Supplementary file 2 [file Table_2.DOCX]

**Supplementary Material S2: Focus Group Interviews**

**Part1** **Focus Group Interviews**

**Title:** Cognitive Interview Guide for a Discrete Choice Experiment (DCE) to Assess Preferences for HPV Self-Sampling Choices

**Introduction:**

Hello and thank you for agreeing to take part in this cognitive interview. Today, we will be discussing a survey questionnaire designed for our study on HPV self-sampling preferences among women in Hohhot.

My role is to facilitate our discussion. The goal of our conversation is to get your feedback on how clear and easy to understand the survey questions are, and to hear about your experience if you were to complete it.

Before we begin, I want to emphasize that your participation is completely voluntary. You are free to pause or end the interview at any point. Everything you share will be kept strictly confidential and will not be linked to your name in any reports. We expect this discussion to last approximately 10 minutes

**Section 1: Background Information**

1. To begin, could you share a bit about yourself, such as your age and the area you live in?
2. Could you tell me whether you have any prior experience with or knowledge of HPV testing or self-sampling?
3. Could you tell me whether you have any understanding of the DCE method?

**Section 2: Comprehension of DCE section's content and filling instructions in the questionnaire** *(Show the participant the attached questionnaire example)*

1. As you look over this section of the questionnaire, how clear are the different attributes (we call them 'attributes') and options (we call them 'levels') of the self-sampling kits? Were any parts confusing?
2. When you see terms like "Moderate accuracy" or "Uncomfortable," what do you think they mean in the context of using a self-sampling?
3. Can you understand how to make choices when filling out the DCE part of the questionnaire? Is the instruction "Please choose the option you prefer" clear? If not, please specify what is confusing.

**Section 3: Burden of filling out the questionnaire**

1. Looking at the number of choice sets (scenarios), how do you feel about it? Does it seem too many, too few, or just right?
2. How long do you think it would take you to fill out a questionnaire like this completely and accurately?
3. Do you find this questionnaire boring and burdensome to answer?

**Closing**

"That covers all the questions I have. I truly appreciate you sharing your thoughts so openly—this feedback is extremely helpful. Before we finish, is there anything else about the survey that stood out to you, either good or bad? Finally, please feel free to reach out if you have any other questions later on. Thank you again for your valuable time and input."

**Precautions:**

1. Ensure sensitivity and respect, as topics like HPV and cancer screening can be personal.
2. Follow up for more details based on the interviewees' responses if needed (e.g., *"Can you tell me more about why that attribute was confusing?"*).

**Part 2 Exemplar Feedback from Focus Group**

Provided here anonymized, illustrative examples of the feedback received during the attribute and level development process for the Discrete Choice Experiment.

**Table 1: Feedback from Focus Group Participants (N=6)**

| **Part. ID** | **Residence** | **Age** | **Key Feedback on Questionnaire Comprehension** |
| --- | --- | --- | --- |
| **FG-01** | Urban | 42 | *"The terms 'Operational Difficulty' and 'Comfort' felt overlapping. It would help if they were explained separately—one about steps, the other about feeling."* |
| **FG-02** | Urban | 29 | *"The price points seem realistic. Having a 'Free' option makes me consider the test more seriously, imagining it as a public health program."* |
| **FG-03** | Urban | 35 | *"The choice task itself was clear after reading the example. I was unsure what 'Moderate accuracy' compared to exactly."* |
| **FG-04** | Rural | 56 | *"I understand 'accuracy' as whether the test is right or wrong. But 'Moderate' is vague. Could you say 'similar to a common test'?"* |
| **FG-05** | Rural | 60 | *"Pictures next to the choices were very helpful. The words for comfort (Comfortable/Uncomfortable) are easy to understand."* |
| **FG-06** | Rural | 28 | *"Nine choices felt acceptable, not too long. I was initially confused about choosing between two kits, but the instructions clarified it."* |
