# Supplementary material for: Women’s preferences for HPV self-sampling in cervical cancer screening: a discrete choice experiment
Source: Front Public Health. 2026 Apr 13;14:1779443. doi: 10.3389/fpubh.2026.1779443 (PMC13111358; doi:10.3389/fpubh.2026.1779443)
Supplement: Supplementary file 3 [file Table_3.DOCX]

**Supplementary Material S3: Example of Questionnaire Version**

**Page 1**

**A Study on the Preferences of Adult Women in Inner Mongolia Autonomous Region for HPV Self-Sampling Testing**

The questionnaire is expected to take approximately 2-4 minutes to complete. After scanning the QR code with WeChat and completing the questionnaire, it will be reviewed by the backend. If approved, you can present the screenshot at the Genetics Department of Inner Mongolia Maternal and Child Health Hospital to collect an HPV self-sampling kit.

Note:

1. Only women may complete the questionnaire.

2. Invalid questionnaires will not be eligible for the kit. Please complete the questionnaire carefully.

**Page 2**

We cordially invite you to participate in our questionnaire survey. Before you begin, please read the following carefully. If you agree to participate, please click “Agree” and begin filling out the questionnaire.

1. Invitation to Participate in This Study.

We cordially invite you to participate in an anonymous online survey (approximately 2–4 minutes long, consisting of 20 questions). This informed consent form provides you with some information to help you decide whether to participate in this survey. Please read it carefully, and if you have any questions, please ask the researcher. Your participation in this study is completely voluntary.

2. Why is this study being conducted?

Through a questionnaire survey, this study aims to investigate the preferences of adult women in Inner Mongolia Autonomous Region regarding HPV self-sampling testing. This information will be used to develop and promote more comfortable and private HPV testing methods, which are of utmost importance in eliminating persistent HPV infections and preventing their progression to cervical cancer.

3. Who will be invited to participate in this study?

The inclusion criteria for this study are:

① Having a clear need or willingness for cervical cancer screening;

② Adult women aged 18–64 in the Inner Mongolia Autonomous Region;

③ Able to clearly understand the meaning of each item in the questionnaire and complete it independently or with the assistance of a statistician.

Exclusion criteria for this study:

① Those who are not voluntarily participating in this study;

② Those with cognitive impairments, mental disorders, or critical illnesses who cannot complete the questionnaire.

4. What are the risks of participating in this study?

Some questions in the questionnaire may make you feel uncomfortable or involve privacy.

5. What are the benefits of participating in this study?

Your responses will help us understand the preferences of adult women in the Inner Mongolia Autonomous Region regarding HPV self-sampling testing, which is of great significance for achieving early diagnosis and treatment of HPV.

6. Is participation in and completion of this study mandatory?

Participation in this study is entirely voluntary. You may refuse to participate or withdraw at any time. We sincerely hope you will agree to participate in this survey.

7. Will my information be kept confidential?

This study will strictly adhere to the provisions of the “Statistics Law of the People's Republic of China” for the management and use of data. The information and materials you provide will be strictly confidential. Research data will be used solely for academic purposes. When research results are published, no personal information will be disclosed, and there will be no adverse effects on you personally. This study has been approved by the Ethics Review Committee of the Inner Mongolia Maternal and Child Health Hospital.

8. Who should I contact if I have questions or difficulties?

If you have any questions related to this study, you may contact the study coordinator (Ms. Ding, contact number: XXX-XXXX-XXXX) at any time.

“I have read this informed consent form. I had the opportunity to ask questions, and all questions have been answered. I understand that participation in this study is voluntary. I may choose not to participate in this study or withdraw at any time by notifying the researcher without facing discrimination or retaliation."

If you agree to and acknowledge the above content, please click the “Agree” button below to begin answering the questions. If you do not agree or acknowledge the above content, please click the “Disagree” button below to terminate this survey.

🗆 Agree and continue

🗆 Disagree and exit

**Page 3**

Cervical cancer is one of the most common malignant tumors of the female reproductive system, and persistent human papillomavirus (HPV) infection is a high-risk factor for the development of cervical cancer. Therefore, timely diagnosis is of utmost importance for eliminating persistent HPV infection and preventing its progression to cervical cancer. Considering privacy and convenience, HPV self-sampling testing may become the mainstream method in the future. Assuming you need to undergo HPV self-sampling testing, there are two self-sampling protocols available before you (Option A and Option B). Except for the listed attributes, all other attributes of Option A and Option B are identical by default. Please select the option you prefer based on your personal circumstances. If you do not prefer either option, you may choose neither.

**Part 1** Below are nine different scenarios. Please make your selections in order.

1. Which of the following two self-sampling schemes would you prefer?


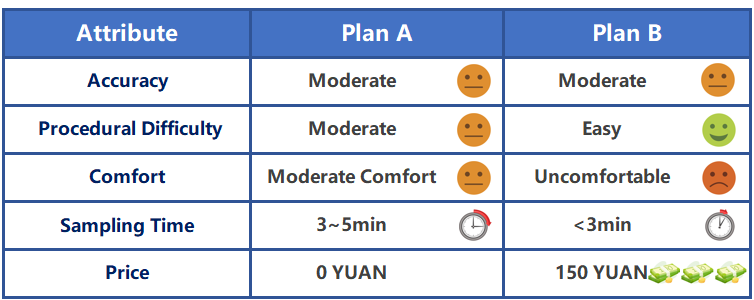


1. Which of the following two self-sampling schemes would you prefer?


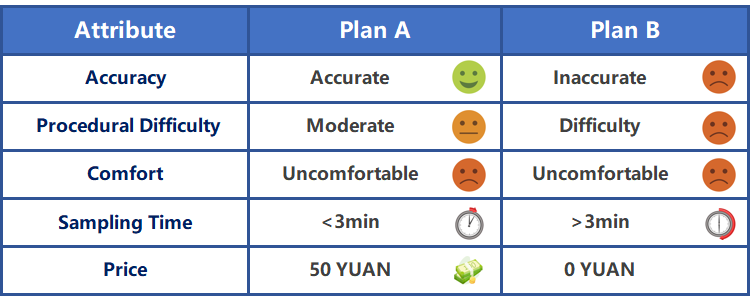


1. Which of the following two self-sampling schemes would you prefer?


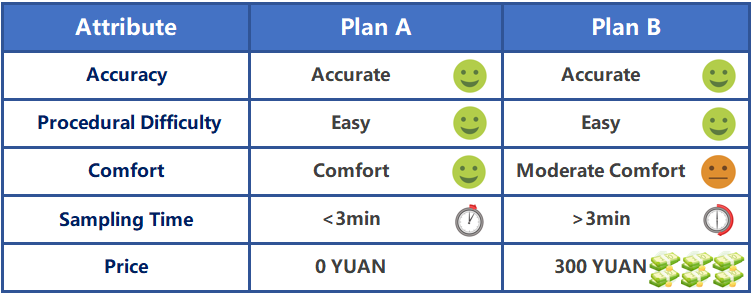


1. Which of the following two self-sampling schemes would you prefer?


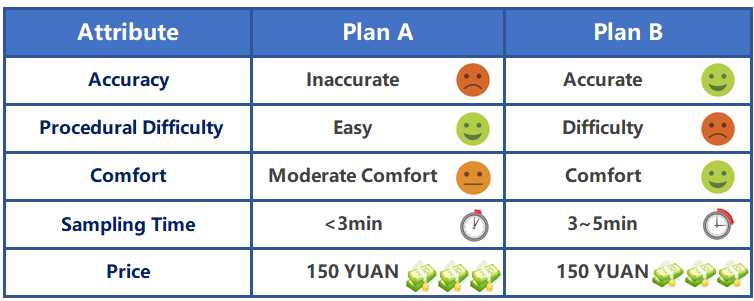


1. Which of the following two self-sampling schemes would you prefer?


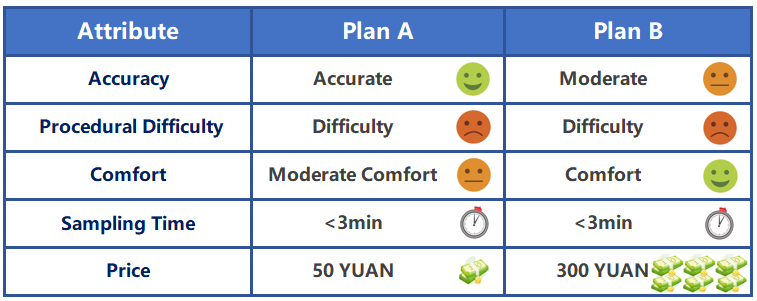


1. Which of the following two self-sampling schemes would you prefer?


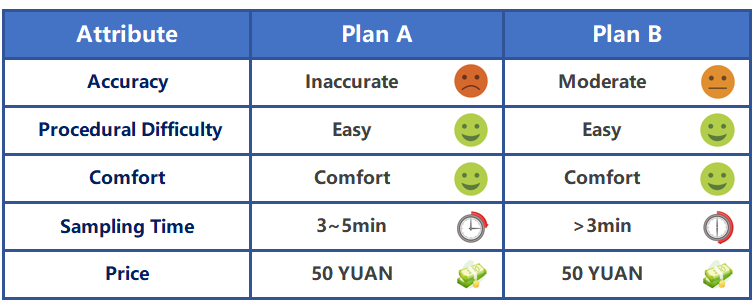


1. Which of the following two self-sampling schemes would you prefer?


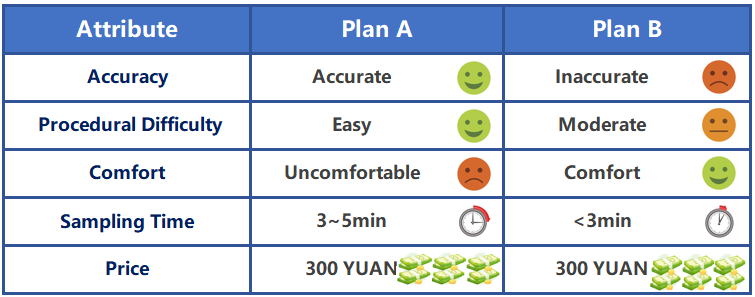


1. Which of the following two self-sampling schemes would you prefer?


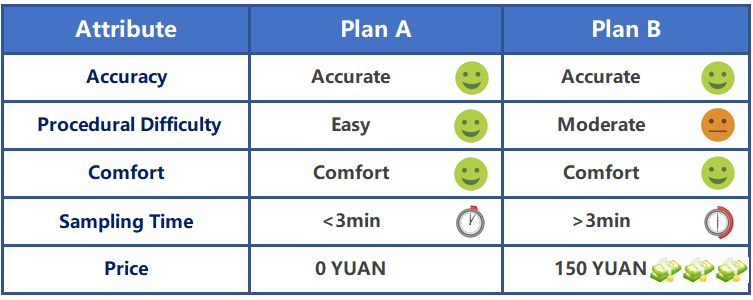


1. Which of the following two self-sampling schemes would you prefer?

**
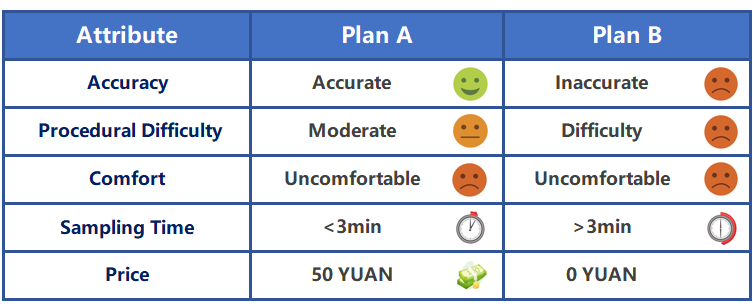
**

*[Question 9 is an attention check questions (identical repeated choice tasks), which is a duplicate of question 2.]*

**Part 2** Please fill in the following questions based on your actual situation.

10. Your gender:

🗆 male🗆 female

11. Date of birth:

12. Your ethnicity:

13. Province, city, and region where you live:

14. Your residence status:

🗆 agricultural household registration

🗆 non-agricultural household registration

15.Your highest level of education (highest degree obtained):

16.Your current career status:

*[Logical jump question: if the condition is met, jump to 16.1]*

---16.1. Your current occupation or occupation prior to retirement:

17. Your family's average monthly income per capita (yuan):

🗆 <=1000 🗆1001-2000 🗆2001-3000 🗆 3001-4000🗆 4001-5000🗆5001-6000 🗆 6001-9000🗆 9001-12000🗆12001-15000 🗆>15000

18. Under conditions where economic, time, and geographical factors permit, do you prefer self-sampling or doctor-administered sampling for HPV testing?

🗆 self-sampling

🗆 Physician sampling

🗆 Mixing and alternating sampling methods

19.You believe that the advantages of self-sampling are:

🗆 Low cost

🗆 Autonomy

🗆 Privacy

🗆 Convenience

🗆 Comfort

🗆 Reduced pressure on medical resources

🗆 Other:

20. Your concerns about self-sampling for HPV testing are:

🗆 Self-sampling is difficult (concerns about inaccurate sampling sites)

🗆 Self-sampling is unsafe (risk of physical injury, etc.)

🗆 Transportation may lead to inaccurate results (contamination, deterioration, expiration, etc.)

🗆 Results are unreliable

🗆 Timeliness of result feedback

🗆 Other:

**Page 4**

Thank you for taking the time to complete this questionnaire.

Wishing you good health!
