# Supplementary material for: Women’s preferences for HPV self-sampling in cervical cancer screening: a discrete choice experiment
Source: Front Public Health. 2026 Apr 13;14:1779443. doi: 10.3389/fpubh.2026.1779443 (PMC13111358; doi:10.3389/fpubh.2026.1779443)
Supplement: Supplementary file 4 [file Table_4.DOCX]

**Supplementary Material S4: DIRECT Checklist**

**Manuscript Title: Women's preferences for HPV self-sampling in cervical cancer screening: a discrete choice experiment**

This document provides the completed DIRECT (Discrete Choice Experiment Reporting Checklist) for the present manuscript. For each item, we have specified the location in the revised manuscript where the required information can be found, along with a brief excerpt of the relevant text.

**Table 1** Checklist for reporting discrete choice experiments in health

| Section Item | Page and paragraph | Relevant Text from Manuscript (Excerpt) |
| --- | --- | --- |
| **Purpose and Rationale** |  |  |
| 1. Describe the real-world context and decision-maker that the hypothetical choice context seeks to replicate or inform. | **Page 3, Paragraph 1; Page 5, Paragraph 4** *(Introduction)* | *“The real-world context is explicitly described in the Introduction. We focus on the Inner Mongolia Autonomous Region, specifically Hohhot, an economically underdeveloped area in Northern China with documented challenges in cervical screening accessibility and coverage. The hypothetical choice context seeks to inform the decisions of the key decision-makers in this setting: under-screened women who are eligible for cervical cancer screening, as stated in the study objective. The DCE simulates the trade-offs these women would weigh regarding HPV self-sampling.”* |
| 2. Provide a rationale for using a DCE to answer the research question. | **Page 6, Paragraph 2** *(Introduction)* | *“DCE was selected for this study because it allows for the systematic elicitation of preferences among attributes... each critical to the design of appropriate and accessible cervical screening programs.”* |
| **Attributes and Levels** |  |  |
| 3. Describe how attributes and levels were derived (e.g. literature review, interviews, focus groups, expert input). | **Page 7, Paragraph 3** *(Methods: Determination of attributes and levels)* | “Firstly, we retrieved previous *literature... Secondly, an expert panel... helped identify additional attributes... Finally, a focus group... was recruited to optimize the expressions for attributes and levels...”* |
| 4. Provide the final list of attributes and levels. | **Page 8, Table 1** | *“The attributes and their levels are detailed in Table 1...”* |
| **Experimental Design** |  |  |
| 5. Report the number of alternatives per choice set and whether they were labelled or unlabeled. | **Page 9, Paragraph 2** *(Methods: Questionnaire development and experimental design)* | *“This design yielded 8 choice tasks, each comprising 2 unlabeled alternatives (described as Option A and Option B) and 1 exit option.”* |
| 6. Describe response options (e.g. forced choice, opt-out, status quo). | **Page 9, Paragraph 2** *(Methods: Questionnaire development and experimental design)* | *“This design yielded 8 choice tasks, each comprising 2 unlabeled alternatives (described as Option A and Option B) and 1 exit option.”* |
| 7. Describe the type of experimental design (e.g. orthogonal, D-efficient, Bayesian efficient, partial profile). | **Page 9, Paragraph 2** *(Methods: Questionnaire development and experimental design)* | *“…an orthogonal main effects design was generated using IBM SPSS Statistics 26.”* |
| 8. Describe which effects are identified in the design (e.g. main effects, higher order interactions, functional form). | **Page 9, Paragraph 2** *(Methods: Questionnaire development and experimental design)* | *“…orthogonal main effects design…”* |
| 9. Describe the number of choice sets, blocks and choice sets per block. | **Page 9, Paragraph 2** *(Methods: Questionnaire development and experimental design)* | *“This design yielded 8 choice tasks, each comprising 2 unlabeled alternatives (described as Option A and Option B) and 1 exit option.”* |
| 10. Indicate how the experimental design was obtained (software, catalogue, other). | **Page 9, Paragraph 2** *(**Methods: Questionnaire development and experimental design)* | *“…generated using IBM SPSS Statistics 26.”* |
| **Survey Design** |  |  |
| 11. Provide a sample choice set and the instructions and background information given to respondents (e.g. providing the survey as an appendix). | **Page 9, Figure 1** (Supplementary Material S3 *- Example of Questionnaire Version)* | *“An example choice task is presented in Figure 1.”* |
| 12. Report any randomization (e.g. choice set order, attribute order, alternative order, framing effects). | **Page 9, Paragraph 2** *(Methods: Questionnaire development and experimental design)* | *“The order of the choice tasks and the appearance of attributes within each task were fixed for all respondents and were not randomized.”* |
| 13. Describe what was checked in piloting (e.g. understanding, respondent burden, timing, wording). | **Page 12, Paragraph 3** *(Methods: Data collection and quality control)* | *“The first ten completed valid questionnaires were treated as an integrated pretest batch. These responses were reviewed for completeness, logical consistency...”* |
| 14. Report whether information from the pilot was used to update the experimental design (e.g. priors, functional form of attributes) or survey design. | **Page 13, Paragraph 1** *(Methods: Data collection and quality control)* | *“Based on these findings, the questionnaire was considered suitable... and no revisions were made...”* |
| **Sample and Data Collection** |  |  |
| 15. Report respondent inclusion/exclusion criteria. | **Page 10 Paragraph 3** *(Methods: Sample Size and Inclusion/Exclusion Criteria)* | *“Inclusion criteria: 1) Female, aged 18 to 64... 2) Have a clear demand... 3) Be able to clearly understand... Exclusion criteria: 1) Involuntary participation... 2) Patients with cognitive impairment...”* |
| 16. Describe how data were collected (e.g. mail, personal interview, web survey). | **Page 11, Paragraph 2** *(Methods: Participant Recruitment)* | *“Trained research assistants were stationed in the hospital's outpatient gynecology departments... Additionally, recruitment posters containing a QR code... were displayed...”* |
| 17. Report the response rate or cooperation rate, if possible. | **Page 15, Paragraph 2** *(Results: Participant Characteristics)* | *“A total of 246 participants completed the questionnaire... of which 200 (81.3%) passed the validity test and were included...”* |
| 18. Report the final sample size and how the sample size was determined. | **Page 10, Paragraph 2** *(Methods: Sample Size and Inclusion/Exclusion Criteria)* | *“Based on this formula, we determine our minimum sample size of N = 125... A target sample size of 125 completes was set.”* |
| 19. Describe respondent characteristics and representativeness of target population, if known. | **Page 15, Table 2** | *“Sociodemographic characteristics of the participants are presented in Table 2.”* |
| **Econometric Analysis** |  |  |
| 20. Indicate coding of data (e.g. effects, dummy, continuous) including definitions. | **Page 13, Paragraph 3** *(Methods: Statistical methods)* | *“In the model specification, econometric analysis employed dummy coding for all categorical attributes...”* |
| 21. Report whether any respondents were removed and why (e.g. suspected fraudulent responses, rationality tests). | **Page 12, Paragraph 2;**  **Page 15, Paragraph 2** *(Methods: Data collection and quality control;*  *Results: Participant Characteristics)* | *“A total of 246 participants completed the questionnaire... of which 200 (81.3%) passed the validity test and were included...”* |
| 22. Provide the rationale for model choice (e.g. conditional logit, mixed logit, latent class) and assumptions (e.g. error variance). | **Page 13, Paragraph 2** *(Methods: Statistical methods)* | *“The analysis employed a mixed logit model, which accounts for preference heterogeneity by allowing attribute parameters to follow random distributions... This approach represents the standard analytical method for DCE data.”* |
| 23. Report model specification. | **Page 13 Paragraph 2** *(Methods: Statistical methods)* | *“U_ji_*_​_ = *β*_0​_ + *β*_1​_*X_1ji​_* + *β*_2​_*X_2ji_*​ + … *+ β_m_X_mji​_* + *ε_ji_”* |
| **Reporting of results** |  |  |
| 24. Report the model performance, goodness of fit (if comparing models). | **Page 17 Paragraph 1** *(Results: Preferences for HPV self-sampling attributes)* | *“The mixed logit model exhibited excellent fit, with a McFadden's pseudo-R² of 0.49. The final model statistics were: LL = -895.57, AIC = 1,829.1, BIC = 1,931.3.”* |
| 25. Describe methods used for analysis of model results (e.g. calculation of marginal rate of substitution, attribute relative importance, welfare gain). | **Page 13, Paragraph 2** *(Methods: Statistical methods)* | *“Willingness-to-pay (WTP) was calculated as the negative ratio of the coefficient for any non-price attribute to the coefficient of the price attribute. The relative importance (RI) of each attribute was calculated by comparing the part-worth utility range for that attribute...”* |
| 26. Report measures of precision for the output(s) of interest (e.g. confidence intervals) and how these were derived. | **Page 13, Paragraph 2** *(Methods: Statistical methods)* | *“Results are presented as coefficients (β) with their standard errors (SE), 95% confidence intervals (CI)…”* |

**References**

1. Ride J, Goranitis I, Meng Y, LaBond C, Lancsar E. A reporting checklist for discrete choice experiments in health: The DIRECT checklist. Pharmacoeconomics [Internet]. 2024;42(10):1161–75. Available from: <http://dx.doi.org/10.1007/s40273-024-01431-6>
